# Maximum surgical blood ordering schedule for elective surgical procedures in Omdurman teaching hospital, Sudan

**DOI:** 10.1186/s12893-024-02458-4

**Published:** 2024-06-01

**Authors:** Mohanad Khalifa, Eman Elhassan, Faisal Ibrahim

**Affiliations:** 1 General Surgery specialist, Sudanese Medical Specialization Board, Khartoum, Sudan; 2 Senior Consultant Urologist, Sudanese Medical Specialization Board, Khartoum, Sudan

**Keywords:** Blood transfusion, Blood in surgery, Cross-match to transfusion ratio (CTR), MSBOS

## Abstract

**Background:**

The need for blood during a surgical procedure is greater than what blood banks are able to provide. There is an excessive amount of blood being ordered for elective surgeries, surpassing the actual requirements. Only 30% of the cross matched blood is actually used in these surgeries. The accuracy of estimating the transfusion needs before a surgical procedure can be determined by looking at the cross match to transfusion ratio and the transfusion index. “These indicators play a crucial role in developing the maximum surgical blood ordering schedule; in this study, these indicators were tested.”

**Aim of study:**

Is to determine the efficiency of blood ordering and transfusion practices for patients undergoing elective surgeries.

**Methods:**

This study is a prospective cross-sectional hospital-based study done at Omdurman Teaching Hospital-Sudan. Conducted for the duration of 6 months period from July to December 2019.The study participants were patients who underwent elective surgical procedures in general surgery and Urology departments as total coverage sample over a period of study duration. Ethical clearance obtained from ethical committee of Sudan Medical Specialization Board.

**Results:**

Two hundreds seven patients included in this study, the amount of blood units requested were 443-unit, cross matching for 98.6% (n 437) of units were done. Only 100 unit were Transfused (22,8%). The calculated CT ratio was 4.4, transfusion index was 1.6 and transfusion probability was 29.9%.

**Conclusion:**

Transfusion probability and transfusion index of the present study were optimal but comparatively higher than the standard guidelines as most of the cross matched blood was not utilized.

## Introduction

The demand for blood during surgical procedures is higher than what blood banks can provide. There is an excessive ordering of blood for elective surgeries, surpassing the actual need [1, 2]. 

The first pre-transfusion cross-matching was conducted in 1907 by Ottenberg [3]. Blood transfusion experiments progressed gradually, starting with animal-to-animal transfusions performed by Richard Lower in 1665 at Oxford, followed by the first animal-to-human transfusion in 1667 by Jean Denis. The first human-to-human blood transfusion took place in 1818 by Blundell. In 1900, Landsteiner classified the ABO blood grouping system [4]. In Sudan, a Blood bank was established within Khartoum Teaching Hospital in the sixties and upgraded to the National Directorate of Blood Bank in 1999 [5]. 

The debate on appropriate blood use started when it was introduced into medical practice. Only 30% of cross-matched blood is actually used during elective surgeries [6]. Variations in transfusion rates are influenced by differences in surgical techniques and skills, lack of transfusion protocols, and preoperative anemia [7]. 

Surgeons, especially trainees, tend to always have cross-matched blood available as a precautionary measure [8]. 

The maximum surgical blood ordering schedule (MSBOS) is a table that lists surgical procedures along with the number of units of blood routinely cross-matched before surgery [1, 7, 8]. 

The accuracy of preoperative estimation of transfusion for a patient undergoing surgery is measured using indicators such as the cross-match to transfusion ratio (CTR) and the transfusion index (TI), which are crucial in developing the maximum surgical blood ordering schedule (MSBOS) [9, 10]. 

## Justifications and rational

According to the World Health Organization (WHO), Sudan is one of 67 countries that reported collecting less than 10 whole blood donations per 1000 population per year in 2013 [11]. Therefore, it is crucial to ensure that the blood that has already been collected is not wasted. One factor contributing to blood wastage is excessive preoperative cross-matching and inefficient utilization [11]. This prompted us to examine how efficiently we are using the blood that has already been collected.

## Objective

The purpose of this study was to assess how effective blood ordering and transfusion practices are for patients who are having planned surgeries.

### Strength and limitations of the study

The strength of this study lies in the fact that there is no known previous similar study conducted locally in Sudan. This provides a unique opportunity to contribute new knowledge to the field. However, a limitation of this study is the lack of medical records in the blood bank, particularly regarding unutilized blood that has been retained from the operating room.

## Literature review

Blood intended for transfusion is deemed safe provided it originates from a meticulously chosen, healthy donor devoid of infections that could pose risks to the recipient. Furthermore, it must undergo rigorous testing, processing, and storage procedures by reliable methods. Transfusion should only occur when necessary and beneficial for the patient’s health and overall well-being.

The blood donation system in Sudan relies on both voluntary and replacement donors [12]. In Khartoum, both replacement and voluntary donation are used to meet the high demand for blood. However, only 40% of the blood collected in Khartoum is from voluntary donors and 60% are from family replacement donors [13]. 

The World Health Organization advises implementing a comprehensive blood transfusion service to ensure safe transfusions. This includes collecting blood from voluntary donors, screening for infections, adhering to good laboratory practices, and minimizing unnecessary transfusions [14, 15]. A Hospital Transfusion Committee acts as a coordinating body to facilitate communication between national transfusion services, hospital blood banks, and clinical staff. It plays a crucial role in establishing policies related to transfusion and addressing any issues that arise [16]. 

The lack of randomized clinical trials in blood transfusion has led to a scarcity of evidence-based protocols. In 1974, Friedman et al. from the University of Michigan utilized the anticipated volume of blood units transfused during hospitalization for typical surgical procedures to establish maximum blood orders for each procedure [1, 17]. 

Various indices have been proposed to evaluate the effectiveness of blood ordering and utilization systems. Boral Henry from the State University of New York introduced the cross-match to transfusion ratio (C/T ratio) [18], while in the 1980s, Mead et al. introduced the concept of transfusion probability (TP). These metrics help assess the efficiency of blood management practices and aid in optimizing blood utilization [17, 19]. 

## Methods

### Study Design, setting and participants

This prospective cross-sectional study was conducted at Omdurman Teaching Hospital (OTH), a tertiary hospital in Sudan, catering to 700–1200 patients daily and performing surgical procedures for approximately 5000 patients annually. Over a six-month period from July to December 2019, the study included patients undergoing elective surgeries in general surgery and urology departments, encompassing the entire patient population meeting the inclusion criteria.

### Inclusion criteria

Adult patients aged 18 years and older who were scheduled for elective surgeries in either general surgery or urology units.

### Exclusion criteria

Individuals not listed in the theatre schedule one day prior to the operation.

The researchers, with the help of junior doctors, collected data through direct observation using patient information sheets. The data was obtained from transfusion services in blood banks, the operating theatre database, and patient files. The variables examined included age, gender, procedure type, number of blood units ordered, number of blood units cross-matched, and number of blood units transfused during the procedure. The collected data was analysed using the Statistical Package for Social Science (SPSS) version 23.0.

Standard metrics such as the cross-match to transfusion ratio (CTR), transfusion probability (%T), and transfusion index (TI) were utilized to assess the suitability of blood ordering and utilization services. Data analysis involved computing simple proportions using the formulas outlined in the original source [20].

**Table:** 

*CTR =*	*Number of units cross –matched* *-----------------------* *Number of units transfused*	A ratio above 2.5 suggests too much blood cross-matching, while a ratio over 2 indicates considerable blood wastage

**Table:** 

*T% =*	*Number of patients transfused×100* *-------------------* *Number of patients cross matched*	A value of < 30 was considered indicative of significant blood wastage

**Table:** 

*T1 =*	*Number of units transfused* *--------------------* *Number of patient’s transfused*	A value of < 0.5 signifies no need for cross-match

The MSBOS was created following Mead’s criterion, which entails multiplying the typical units of blood utilized in a procedure by 1.5 to ascertain the quantity of blood units necessitating preoperative cross-matching for each procedure. This method mirrors approaches utilized in comparable studies [18, 20–22].

### Ethical considerations

Ethical considerations were considered in this study. The hospital gave consent and provided written permission for the collection of information, which was done anonymously to ensure data confidentiality. Additionally, ethical clearance was obtained from the ethical committee of the Sudan Medical Specialization Board.

## RESULTS

Six hundred and thirty-four patients underwent elective surgical procedures in the general surgery and urology departments. Of these, 87.2% (*n* = 553) underwent procedures in the general surgery department, while 12.8% (*n* = 81) were treated in the urology department. The predominant age group was between 26 and 40 years, with 54% of the study population being female.

**Fig. 1:**
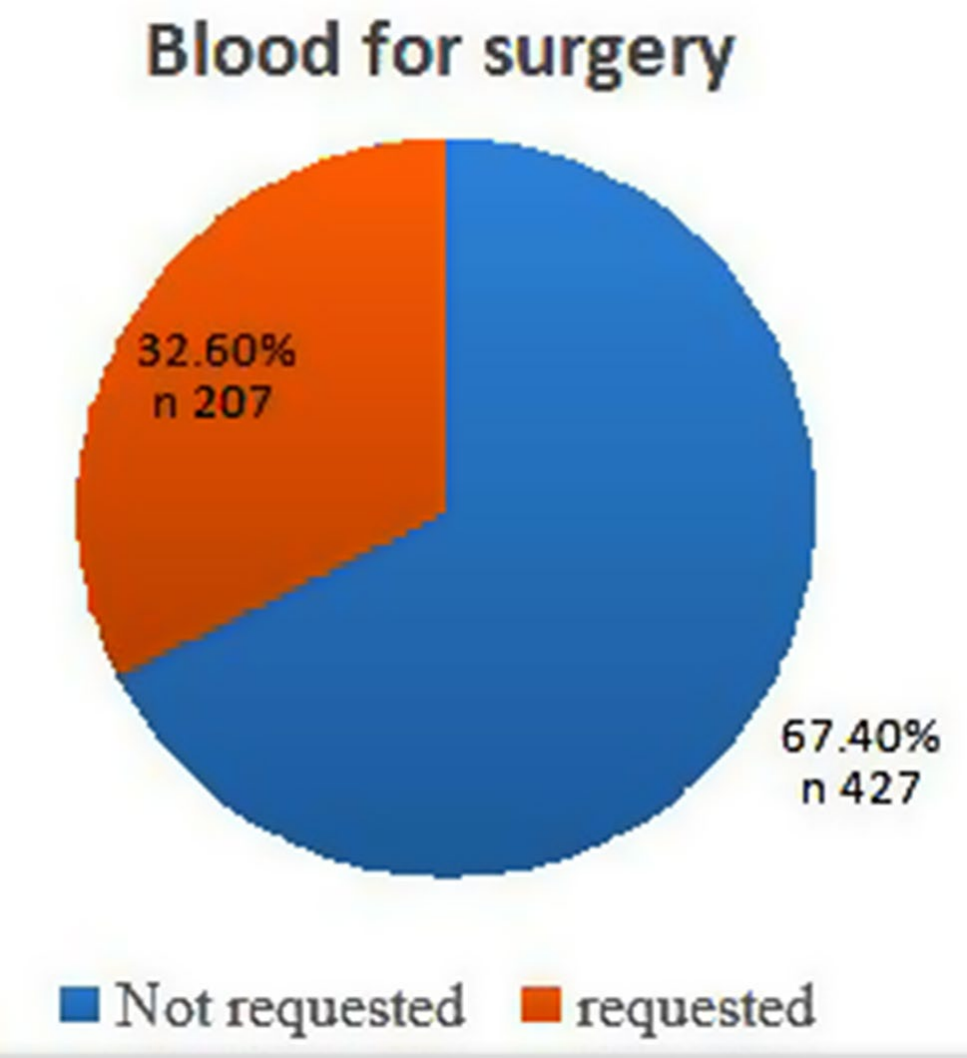
The frequency distribution number of blood requests

Four hundred twenty-nine patients underwent surgical procedures without blood preparation, accounting for 67% of the total number of patients (Fig. 1). However, the data of 207 patients with blood prepared one day before surgery were used to calculate the MSBOS, according to the inclusion criteria of this study.

Out of the 207 patients, 443 units of blood were requested, with cross matching done for 98.6% (*n* = 437) of these units. The procedures that required the highest number of blood units were cholecystectomy (122 units) and thyroidectomy (74 units). The maximum number of blood units requested for a single procedure was 6 units, which was for splenectomy. Table (1).

**Table 1:** Comparison between number of patients, blood units requested, cross matched and transfused

Procedure/ diagnosis	No. of Patients	Blood Unit requested	No. of Cross-matched blood unit	No. of Patients transfused	No. of units transfused
Incisional hernia	19	39	38	8	12
Cholecystectomy	61	121	117	4	5
CBD exploration	6	12	12	1	2
Thyroidectomy	37	74	74	10	12
Mastectomy	19	39	38	9	16
APR	1	4	4	1	4
Bowel resection and anastomosis	9	18	18	5	8
Colostomy	5	10	10	1	1
Reversal of colostomy	3	6	6	2	3
Feeding tube	2	3	3	0	0
Splenectomy	4	22	22	3	8
Parotidectomy	1	2	2	0	0
Whipple procedure	1	4	4	1	2
Elective Amputation	4	8	8	3	4
Nephrectomy	7	24	24	5	12
Nephrolithotomy	7	14	14	2	2
Ureterolithotomy	4	8	8	0	0
TVP	4	8	8	3	4
Pyeloplasty	2	4	4	0	0
URS	4	8	8	0	0
TURP	3	7	7	1	2
TURBT	4	8	8	3	3
Total	**207**	**443**	**437**	**62**	**100**

**Fig. 2:**
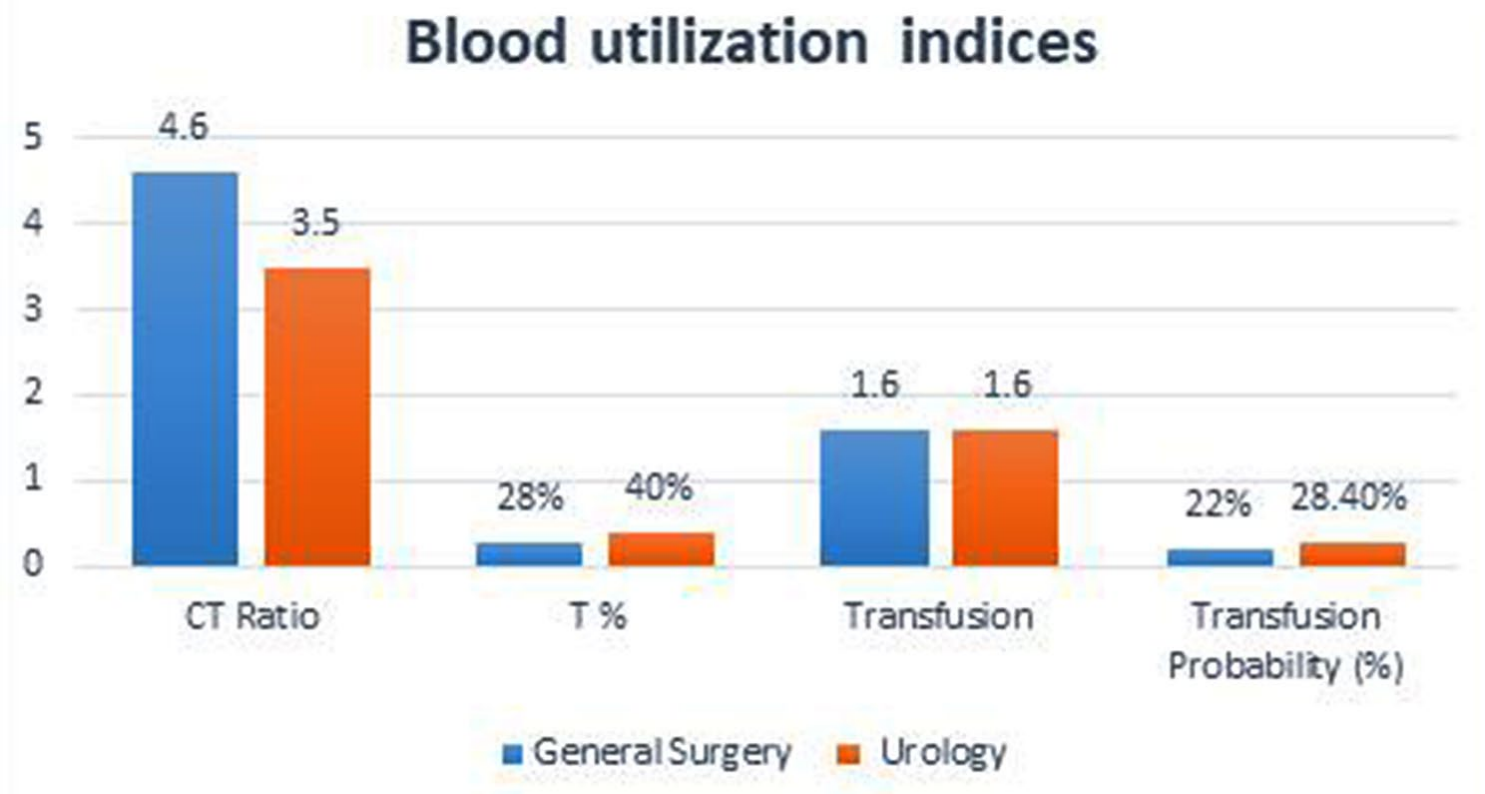
Utilization indices for patients in general surgery and urology

The calculated CT ratio was 4.4, transfusion index was 1.6 and transfusion probability was 29.9%. Utilization indices for patients in general surgery and urology showed in (Fig. 2). Moreover, (Table 2) shows the calculated CT ratios, transfusion index, transfusion probabilities and blood utilization for various surgical and urological procedures.

The highest CT ratio and lowest transfusion probability was calculated for Cholecystectomy 23.4 procedures followed by Colostomy 10 and Nephrolithotomy 7, while APR had the lowest CT ratio one.

The transfusion probability (%T) for the general surgery was 28% and 40% for urology, and that for all patients requiring cross match was 29.9%. Among the general surgery patients, patients undergoing Whipple procedure and APR had highest %T of 100% while in urology was 75% for TURBT and 74% for TVP.

The highest transfusion index (TI) was observed in APR (4), followed by Splenectomy (2.6) and Nephrectomy (2.4). Surgical procedures with the lowest TI included surgical feeding tube insertion, parotidectomy, ureterolithotomy, and URS, all with a TI of zero.

**Table 2:** Blood utilization indices for General surgery and Urology procedures

Procedures/ diagnosis	CT Ratio	T %	Transfusion Index	Transfusion Probability (%)
Incisional hernia	3.17	42.10%	1.5	31.60%
Cholecystectomy	23.4	6.60%	1.25	4.27%
CBD exploration	6	16.70%	2	16.70%
Thyroidectomy	6.17	27.00%	1.2	16.20%
Mastectomy	2.3	47.40%	1.7	42.10%
APR	1	100.00%	4	100.00%
Bowel resection and anastomosis	2.25	55.60%	1.6	44.40%
Colostomy	10	20.00%	1	10.00%
Reversal of colostomy	2	66.70%	1.5	50.00%
Surgical Feeding tube			0	
Splenectomy	2.7	75.00%	2.6	36.40%
Parotidectomy			0	
Whipple procedure	2	100.00%	2	50.00%
Elective Amputation	2	75.00%	1.3	50.00%
Nephrectomy	2	71.40%	2.4	50.00%
Nephrolithotomy	7	14.00%	1	28.60%
Ureterolithotomy			0	
TVP	2	74.00%	1.3	50.00%
Pyeloplasty			0	
URS			0	
TURP	3.5	33.30%	2	28.60%
TURBT	2.7	75.00%	1	37.50%

Based on transfusion probabilities, the Mead’s criterion was used to calculate the maximum surgical blood order schedule (MSBOS), which is determined by multiplying the TI by 1.5. The calculated MSBOS for each procedure is provided in (Table 3). Abdominoperineal resection (APR) had the highest number of blood units scheduled. For surgeries with a TI of zero, a type and screen policy were proposed.

**Table 3:** Calculated MSBOS for each procedure

Diagnosis/ procedure	TI	Calculation	Proposed MSBOS	(Blood units) Approximately
Incisional hernia	1.5	1.5 × 1.5	2.25	2
Cholecystectomy	1.25	1.25 × 1.5	1.9	2
CBD exploration	2	2 × 1.5	3	3
Thyroidectomy	1.2	1.2 × 1.5	1.8	2
Mastectomy	1.7	1.7 × 1.5	2.5	2–3
APR	4	4 × 1.5	6	6
Bowel resection and anastomosis	1.6	1.6 × 1.5	2.4	2
Colostomy	1	1 × 1.5	1.5	2
Reversal of colostomy	1.5	1.5 × 1.5	2.25	2
Feeding tube	0	0 × 1.5	0	G&S
Splenectomy	2.6	2.6 × 1.5	3.9	4
Parotidectomy	0	0 × 1.5	0	G&S
Whipple procedure	2	2 × 1.5	3	3
Elective Amputation	1.3	1.3 × 1.5	1.95	2
Nephrectomy	2.4	2.4 × 1.5	3.6	3
Nephrolithotomy	1	1 × 1.5	1.5	2
Ureterolithotomy	0	0 × 1.5	0	G&S
TVP	1.3	1.3 × 1.5	1.95	2
Pyeloplasty	0	0 × 1.5	0	G&S
URS	0	0 × 1.5	0	G&S
TURP	2	2 × 1.5	3	3
TURBT	1	1 × 1.5	1.5	2

## Discussion

The study conducted at Omdurman Teaching Hospital aimed to assess the blood ordering practices of the general surgery and urology departments over a six-month period, with the goal of optimizing blood transfusion practices and preventing unnecessary transfusions. One strength of the study is its novelty, as there were no similar studies conducted locally in Sudan, providing an opportunity to contribute new knowledge to the field. However, a limitation was the lack of medical records in the blood bank, particularly regarding unutilized blood retained from the operating room.

The study found that a large portion of cross-matched blood units, around 77%, weren’t used, showing inefficient blood use. This matches findings from similar studies by Vibhute et al. and Basnet et al. [23, 24] Different procedures had varying chances of needing a transfusion. Importantly, the ratio of cross-matched blood to actual transfusions (CTR) was higher than recommended [25–31], indicating too much blood was ordered but not used. The overall CTR of 4.4 in this study was higher than recommended, suggesting wasteful blood use. Possible reasons include doctors ordering more blood than needed, possibly due to caution, rather than following guidelines.

The study also evaluated transfusion probability (%T) and transfusion index (TI) to assess blood transfusion practices. The results indicated inappropriate transfusion practices and inefficient blood utilization, with TI values far from the standard [28]. Procedures with a TI of zero were scheduled for blood grouping and screening, following the “group and screen” policy, known to reduce unnecessary cross-matching of blood effectively [32]. 

In comparison to other studies [27, 33], the overall transfusion probability (%T) calculated in this study was lower than the recommended threshold of 50%, suggesting inappropriate transfusion practices. Additionally, the transfusion index (TI) values were higher than the standard, indicating inefficient blood usage [32, 34–37]. These findings highlight the need for optimizing blood transfusion practices to ensure efficient utilization and minimize unnecessary cross-matching of blood units.

The study recommends implementing Maximum Surgical Blood Order Schedule (MSBOS) to address the high cross-match to transfusion ratio (CTR) and improve blood utilization. However, strict adherence to MSBOS within hospitals may be challenging due to the variability in predicting blood loss during surgery. Nonetheless, with combined efforts from surgeons and hospital transfusion services, the rationalization of blood ordering practices and implementation of MSBOS can lead to more efficient blood utilization and improved patient care.

The effectiveness of MSBOS protocols is influenced by local circumstances, clinical practices, and patient variables, suggesting the need for tailored approaches and regular review. Implementing MSBOS can be challenging due to the unpredictability of blood loss during surgery, but collaboration between surgeons and hospital transfusion services can enhance its success. Further research into factors like preoperative anemia and surgical techniques is crucial for improving blood management practices and patient outcomes.

## Conclusion

The blood transfusion indicators in our study were found to be optimal, but they were relatively higher than the standard guidelines since a significant portion of the cross-matched blood was not actually used. This discrepancy highlights the need for further investigation into factors influencing blood usage, such as surgical techniques and patient’s factors.

## Data Availability

The data are available at the medical records of blood bank and Operation Theater at Omdurman Teaching Hospital, Omdurman, Sudan.For more Data that support the findings You Can contact author at this e-mail: nadkhalifa33@gmail.com.
